# The hatching larva of the priapulid worm *Halicryptus spinulosus*

**DOI:** 10.1186/1742-9994-6-8

**Published:** 2009-05-26

**Authors:** Ralf Janssen, Sofia A Wennberg, Graham E Budd

**Affiliations:** 1 Uppsala University, Department of Earth Sciences, Palaeobiology, Villavägen 16, 75236 Uppsala, Sweden

## Abstract

Despite their increasing evolutionary importance, basic knowledge about the priapulid worms remains limited. In particular, priapulid development has only been partially documented. Following previous description of hatching and the earliest larval stages of *Priapulus caudatus*, we here describe the hatching larva of *Halicryptus spinulosus*. Comparison of the *P. caudatus *and the *H. spinulosus *hatching larvae allows us to attempt to reconstruct the ground pattern of priapulid development. These findings may further help unravelling the phylogenetic position of the Priapulida within the Scalidophora and hence contribute to the elucidation of the nature of the ecdysozoan ancestor.

## Background

**Figure 1:**
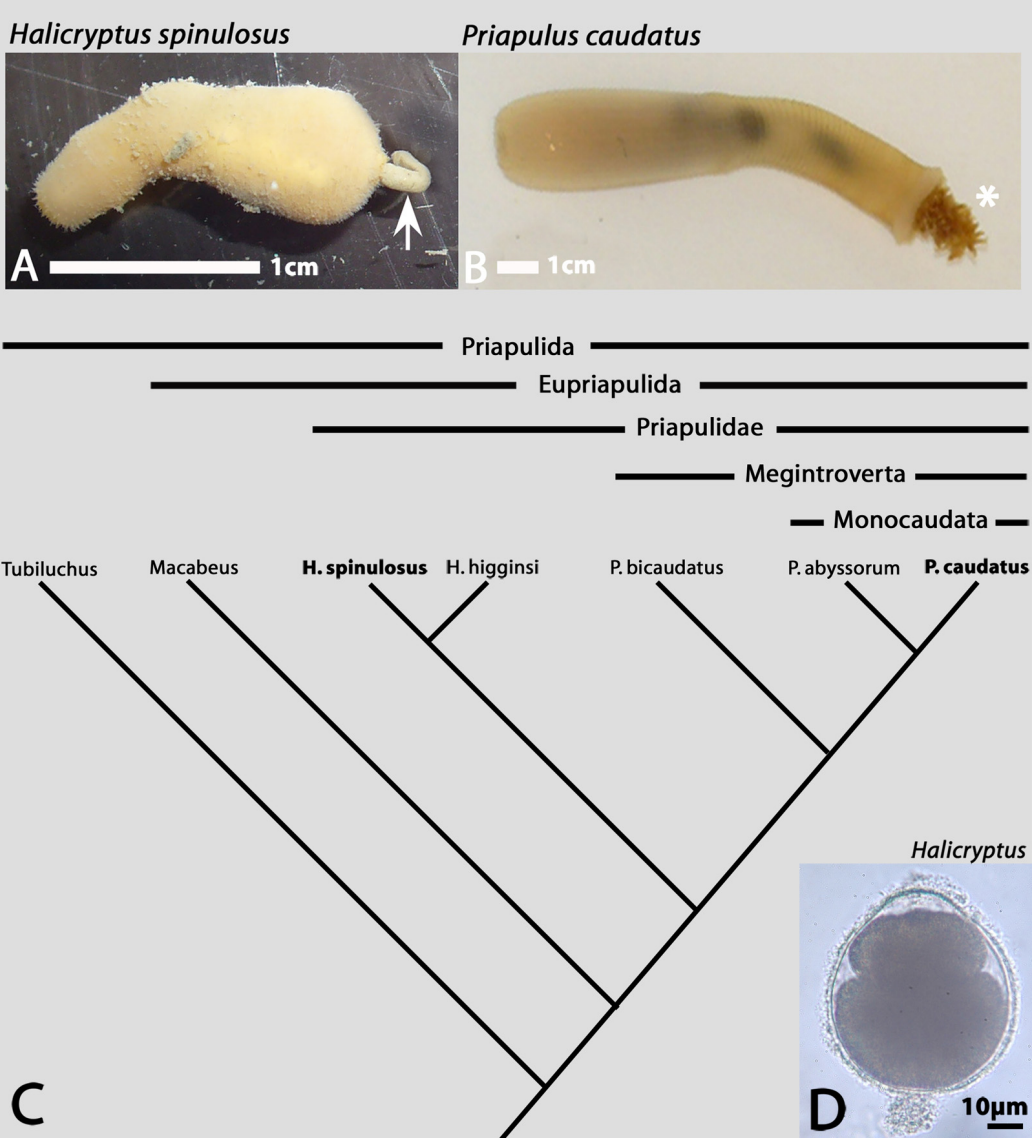
**A/B Adults with fully protruded introvert of the priapulid worms *Halicryptus spinulosus *(A) and *Priapulus caudatus *(B)**. Anterior is to the left in both cases. The posterior structure in A (arrow) is excrement, not a caudal appendage as seen in B (asterisk). **C **Simplified phylogeny of the Priapulida (after [19]). **D **Embryo of *H. spinulosus *one day before hatching. View on inner organs like the developing gut is inhibited by large amounts of yolk. Anterior is to the top.

The Cycloneuralia is a small group of worms, poorly-known apart from the numerous and important nematodes. With the advent of the Ecdysozoa theory [1] they came to be considered as the sister group to the Arthropoda, the most diverse and abundant living animal clade. Within the Cycloneuralia, the Priapulida have been placed basally by some analyses; possibly even within the Ecdysozoa as a whole, leading to them being sometimes described as "living fossils" [1-4]. Despite their obviously important phylogenetic position, information on their biology is scarce – especially concerning their embryonic development and their earliest larval stages. Even data on the best-studied priapulid, *Priapulus caudatus*, the priapulid species whose genome was recently approved to be sequenced , was until recently scarce, incomplete and contradictory [3-8]. Particular interest in scalidophoran and priapulid larvae has increased with the finding of fossilized putative stem group scalidophoran embryos from the Cambrian [9,10]. A proper comparison of such fossil embryos with recent embryos/larvae is obviously only possible if the corresponding embryonic stages and larvae can be described in detail. In our recent work we described for the first time the confirmed earliest larval stages of *P. caudatus*, which possessed some surprising features, such as the lack of a mouth. At that time it was unclear whether these features were apomorphic for *P. caudatus *or were plesiomorphic within Priapulida [4]. Here we describe the hatching larva of the distantly related priapulid worm *Halicryptus spinulosus *(Fig. 1A–C) and compare it with the hatching larva of *P. caudatus *[4].

## Results

Although many thousands of eggs could be obtained and fertilized in this study, the success rate of development to hatching was extremely low, with the result that only 52 specimens of hatched larvae were available for this study. This is in contrast to a previous study of *P. caudatus *where many tens of thousands of hatching larvae and large numbers of subsequent larval stage specimens could be obtained under controlled laboratory conditions. This disparity is likely linked to sperm quality. As measured by sperm motility, the quality of *H. spinulosus *sperm was much lower than for *P. caudatus*: only very few spermatozoa swam actively and were separated from the gonads and/or sister-spermatozoa; possibly leading to delayed fertilization or polyspermy (if the eggs were fertilized by floating or swimming "groups" of attached spermatozoa). Because of this restriction we were not able to examine all documented features in great numbers. Nevertheless, the stability of the results obtained strongly suggests that the characters we describe are natural features of the *H. spinulosus *hatching larva.

The first *H. spinulosus *larva hatched 21 days after fertilization when kept at approximately 4°C. In the following three days the remaining larvae hatched implying that the development is largely synchronized. Most of the obtained larvae (40) were processed for Scanning Electron Microscopy (SEM), 12 were allowed to develop in order to reach subsequent larval stages. Although these latter hatching larvae were alive for several weeks they did not moult and eventually died.

**Figure 2:**
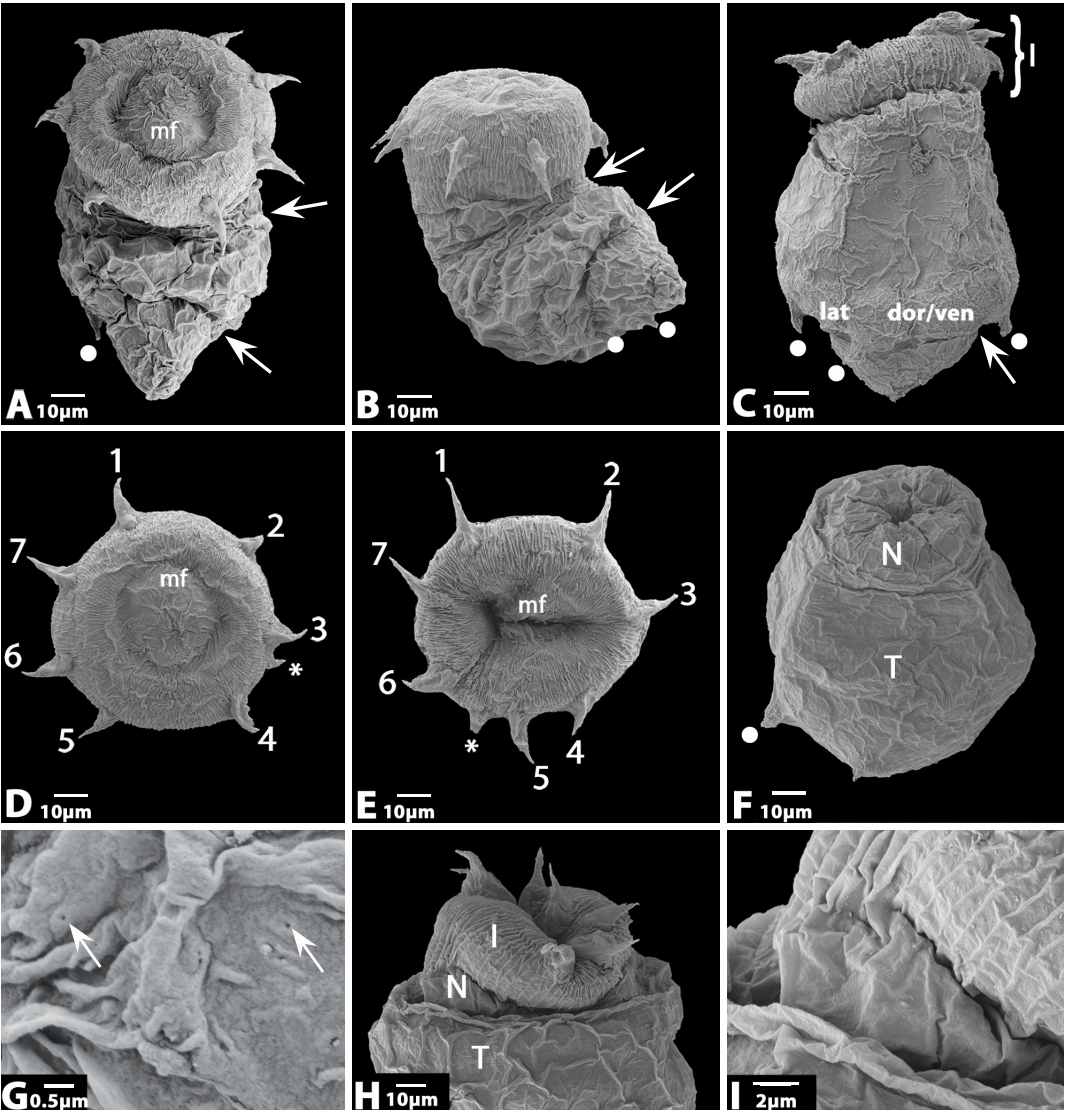
**A Hatching larvae with everted introvert**. Anterior up. Arrows point to folds caused by movement of the larvae. Dot denotes posterior tubulus. **B **Same larva as in A rotated by 90°. Anterior up. Dots and arrows as in A. **C **Another hatching larva. Anterior up. Three tubuli are visible (dots). Bracket indicates the introvert. Arrow as in A **D **Anterior view of larva shown in A and B. Seven first-ring scalids and one smaller second-ring scalid (asterisk). **E **Anterior view of another larva. **F **Larva with fully retracted introvert. Anterior up. Dot as in A. **G **Magnification of the cuticle surface of the mouth field (mf). Arrows point at small pores. **H **Anterior part of a hatching larva with protruded introvert. Anterior up. Introvert (I), neck (N) and trunk (T) are clearly distinguishable. **I **Magnification of the larva shown in H. Abbreviations: 1–7, first-ring scalids one to seven; dor/ven, dorsal or ventral; I, introvert; lat, lateral; mf, mouth field; N, neck; T, trunk.

**Figure 3:**
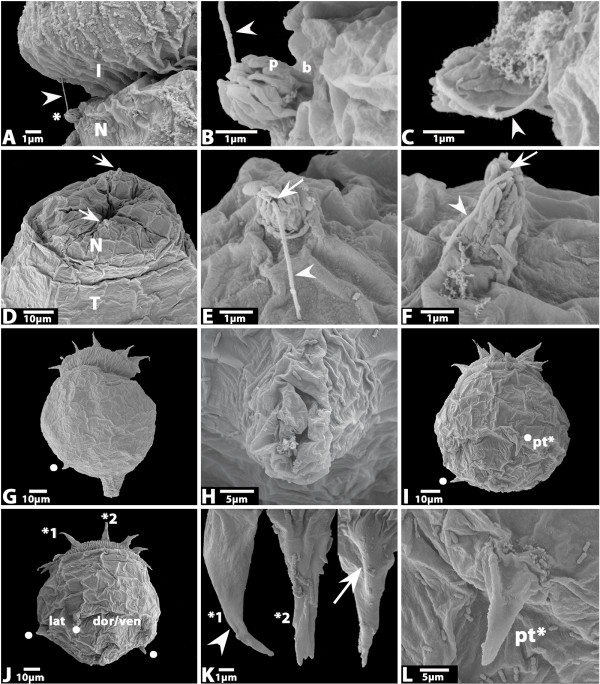
**A Cut-out showing the neck region of a larva with a flosculus (asterisk) situated at the transition of introvert (I) to neck (N)**. Arrowhead points to filiform structure emanating from the terminal pore of the flosculus. **B **Magnification of the flosculus seen in A. Arrowhead as in A. **C **Flosculus found on another not shown larva. Arrowhead as in A. **D **Magnified anterior part of the larva shown in Fig. 2F. Anterior is up. Arrows point to the two flosculi at opposite sites of the larva. **E/F **Magnification of the lower (E) and upper (F) flosculus shown in D. Arrows point to terminal pores. Arrowheads as in A. **G **Larva with prominent protrusion at the rear end. Anterior is up. Dot marks posterior tubulus. **H **Magnification of the protrusion shown in G from a different angle. **I **Larva with two visible posterior tubuli (dots). **J **Larva with three visible posterior tubuli (dots). Nearby tubuli mark lateral. Position of distant tubuli mark ventral and dorsal respectively. **K **Three first-ring scalids, two of which are seen in situ in J marked by numbered asterisks. The third (right) belongs to the larva shown in Fig. 2A/B. Lateral (left) view, ventral (middle) view and dorsal (right) view. Note that the scalid in the middle is with a branched tip, the others are with a single tip. Arrow marks dorsal ridge. Arrowhead points to a micro-spine. **L **Magnification of the tubulus marked with "pt*" in I. Abbreviations as in Fig. 2; b, base of flosculus; p, papillae; pt, posterior tubulus.

The lack of a proper lorica is the most obvious feature of the *H. spinulosus *hatching larva (Figs 2A–C, F and 3G, I, J); a feature also lacking in *P. caudatus *[4]. Since a lorica is present in all hitherto described larvae of *H. spinulosus *it is clear that the hatching larva has never been described before [11-21]. The assumption of Purasjoki [13] that he must have described the first larval stage because of its similar size (290 μm and 340 μm) as the "mature egg" (280 μm) is clearly wrong, not only because the larva described here is much smaller, but also because the size of the egg is obviously less than 280 μm. In figure 1D we show an egg with an almost fully developed embryo. The eggs are completely round and an anterior extension as described by Purasjoki [13] was never observed. The size of the eggs (ca. 50–60 μm) and the hatching larva with fully protruded introvert (ca. 120 μm) is approximately the same as for *P. caudatus *(cf. [3,4]). The *H. spinulosus *hatching larva also superficially resembles the hatching larva of *P. caudatus *in its light-bulb shaped appearance (cf. [4]). However, an obvious difference concerns the motility of the larvae. Whereas the *P. caudatus *hatching larva is only capable of moving its introvert in and out, that of *H. spinulosus *is also able to bend its complete body – possibly because of an extra set of muscles and/or (at least) two horizontal lines of weaknesses in the cuticle covering the trunk (Fig. 2A, B). The hatching larvae of *P. caudatus *and *H. spinulosus *both lack a mouth and pharyngeal teeth (Fig. 2A, D); and share the arrangement of 7+1 scalids (Fig. 2D, E), the subdivision of the body into introvert, neck and trunk (Fig. 2C, H, I) and the presence of four weakly developed posterior tubuli of which maximal three are seen on the photographs presented here (Figs 2A–C, F and 3G, I, J) (cf. [4]). In some cases a few micro-spines (<1 μm) are sparsely distributed on their surface (Fig. 3L and not shown). The tubuli are arranged in pairs of two with the pairs of closer together tubuli being situated at the putative lateral sides of the larva, as also shown for later larval stages and larvae of other priapulids (Figs 2C and 3J) (e.g. [4,14]). In later larval stages four tubuli are also present, but they are located only slightly below the midregion of the lorica (trunk) on the lateral plates [14]. The arrangement of the scalids in an anterior ring armed with seven first-ring scalids and one to three somewhat more posteriorly-located smaller second-ring scalids is less clear than for *P. caudatus *(cf. [4]). In fact we only found larvae with one secondary scalid (Fig. 2D, E). With exception of the size first-ring (12–14 μm) and second-ring scalids (6–10 μm) were identical. The scalids are thus considerably shorter than in *P. caudatus *(cf. [4]). They have a triangular basis emanating from the introvert, a prominent dorsal ridge, are pointed backwards (when the introvert is protruded) and bend towards the body (Fig. 3K). The tip is branched or with a single tip (Fig. 3K). Occasional micro-spines (<1 μm) are present on some of the primary scalids (Fig. 3K). The surface of the introvert is characterized by a number of vertical ridges (Fig. 2H, I), similar as in adults. The surfaces of the neck and trunk are smooth. The surface of the cuticle spanning the anterior of the larva that will in later stages be armed with pharyngeal teeth is covered with small pores (Fig. 2G). These small irregularly allocated pores, approximately 60 nm in diameter, may represent openings of sensory organs.

The *Halicryptus *hatching larva possesses two outgrowths, the neck-flosculi, on opposite sites of the body, at the border between introvert and neck (Fig. 3A–F). They are 1–2 μm long and composed of a short base and circlets of cuticular papillae at the tip that surround a single pore (Fig. 3B, E, F). Interestingly all the neck-flosculi in the *H. spinulosus *hatching larva are associated with a filiform structure (ca. 3 μm long) emanating from the centre of each flosculus (Fig. 3A–F). The nature and function of these structures is unclear, although the flosculi themselves are clearly sensory organs [18]. In older larval stages of *H. spinulosus *the number of neck-flosculi increases to at least 15 [18]. For the *P. caudatus *hatching larva we described two outgrowths in a similar position [4]. These represent tubular structures (neck-tubuli) rather than flosculi. It is unlikely that the ca. 6.5 μm long neck-tubuli in *P. caudatus *are homologous to the flosculi of *H. spinulosus *since flosculi in general were never reported for any described *P. caudatus *larva. One exception is the documentation of a single flosculus-like structure on a putative early larva of *P. caudatus *[22], but if this larva actually represents a larva of *P. caudatus *is unclear (discussed in [4]).

In a few fixed larvae the rear end formed a tapered structure possibly representing the anal region (Fig. 3G, H). Whether or not a proper anal opening is present is unclear. However its presence is unlikely because of the lack of a mouth and the reported lack of an anus in the *P. caudatus *hatching larva (cf. [4]). The hatching larvae of both *P. caudatus *and *H. spinulosus *are still yolk-rich and as discussed for the *P. caudatus *larva, it seems likely that the *H. spinulous *hatching larva simply digs down into the mud in order to escape from the dangerous surface of the seafloor.

The systematics of the priapulids are not well-characterised. However, Lemburg [19] places both *Priapulus *and *Halicryptus *within the Priapulidae, although distant from each other, a position supported by both Dong et al. [9] and Wills [23]. Their shared developmental data thus allows a partial reconstruction of the ground plan of the Priapulidae hatching morphology (Fig. 1C): It includes the following features: 1) of light-bulb shape, 2) subdivided into introvert, neck and trunk, 3) lacking a true lorica, 4) without a mouth, 5) without pharyngeal teeth, 6) armed with 7+X scalids and 8) with four posterior tubuli. Further investigation of more basal taxa such as *Tubiluchus *will be required to extend the investigation of ground plan development to the Priapulida as a whole, an essential prelude to phylogenetic reconstruction of their morphological and developmental evolution [24].

## Methods

### Halicryptus spinulosus breeding conditions

We fertilized apparently mature eggs of *Halicryptus spinulosus *during several field seasons from winter 2007 to early spring 2009. Although in all cases a number of eggs were successfully fertilized as recognized by the formation of a fertilization-membrane and the commencement of cleaving, hatched larvae were only been observed once (early winter 2008). In all other cases fertilized eggs underwent irregular cleavage (not shown) and eventually died before reaching the larval stage. Priapulid collection, preparation, fertilisation and breeding were performed in the same way during all of the field seasons. Sexually mature female and male specimens of *H. spinulosus *were collected from Trosa archipelago close to the Askö marine station on the east coast of Sweden south of Stockholm. Gonads were stripped off and eggs were artificially fertilised as described for *Priapulus caudatus *[3,4]. The developing eggs were rinsed every day with fresh filtered see water collected from the same collection site as the adults. Decaying eggs where removed. To maintain water quality throughout the developing phase water was frozen after filtration and thawed the day before use. Embryos and hatching larvae were kept at approximately 4°C in a conventional fridge.

### Larvae fixation and preparation

To ensure that the introvert was protruded a needle was used to mechanically push it out from the rear. The hatching larvae were fixed either in 2.5% glutaraldehyde for four hours at room temperature or over night at 4°C in the fridge. Storage and preparation for Scanning Electron Microscopy (SEM) was performed as described in [4].

### Documentation techniques

For SEM pictures, a Zeiss Supra 35VP scanning electron microscope with field emission gun was used. For light microscopic pictures, a Nikon D70 camera was attached to a Nikon Eclipse E400 light microscope. Further processing of SEM and light microscopy images was carried out in Photoshop CS.

## Competing interests

The authors declare that they have no competing interests.

## Authors' contributions

RJ set up the *Halicryptus *project. He was mainly responsible for collection and breeding. He prepared the tables and wrote the manuscript.

SAW contributed to the collection and breeding. She was responsible for preparation of the material and taking SEM photographs.

GEB is the head of the group. He initiated the priapulid project this study is part of, and contributed to the discussion and writing the manuscript.
